# Supplementary material for: A synthetic RNA editing factor edits its target site in chloroplasts and bacteria
Source: Commun Biol. 2021 May 10;4:545. doi: 10.1038/s42003-021-02062-9 (PMC8110955; doi:10.1038/s42003-021-02062-9)
Supplement: Supplementary file 2 — Supplementary Information [file 42003_2021_2062_MOESM2_ESM.pdf]

Supplementary Information

A synthetic RNA editing factor edits its target site in chloroplasts and bacteria

Santana Royan[1], Bernard Gutmann[2], Catherine Colas des Francs-Small[2], Suvi Honkanen[2,3], Jason Schmidberger[1], Ashley Soet[1], Yueming Kelly Sun[2], Lilian Vincis Pereira Sanglard[2], Charles S. Bond[1], Ian Small[2]

[1]School of Molecular Sciences, The University of Western Australia, Crawley 6009, Australia
[2]Australian Research Council Centre of Excellence in Plant Energy Biology, School of Molecular Sciences, The University of Western Australia, Crawley 6009, Australia
[3]Synthetic Biology Future Science Platform, CSIRO, Australia

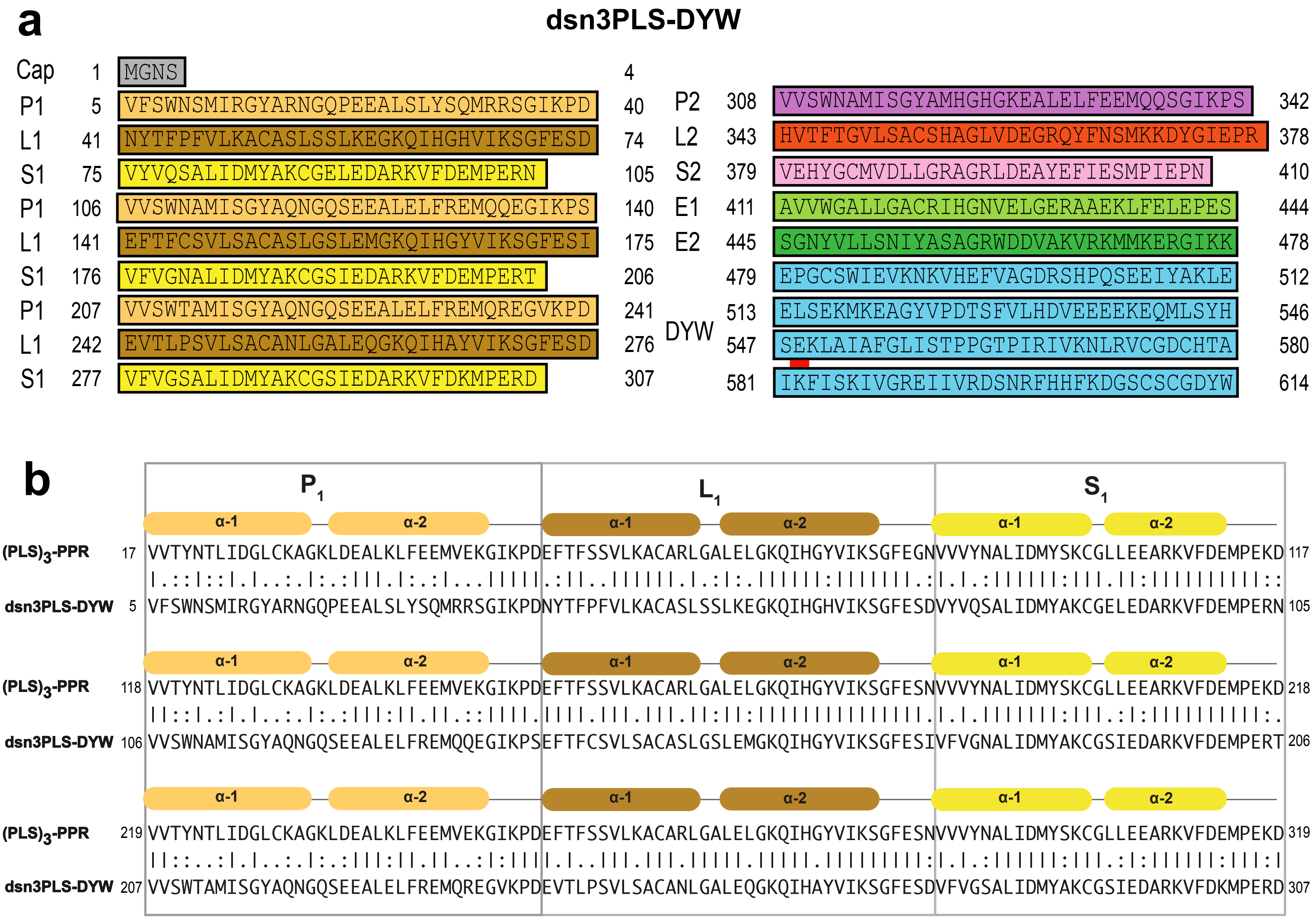

Supplementary Figure S1. Comparison of the sequences of dsn3PLS-DYW and (PLS)3-PPR.
a: Sequence and motif structure of dsn3PLS-DYW. The catalytic E70 residue within the DYW domain is underlined in red. **b**: Alignment of the $(P1L1S1)_3$ regions of dsn3PLS-DYW and $(PLS)_3$-PPR[1]. Overall, throughout this region the two synthetic proteins are only 64% identical.

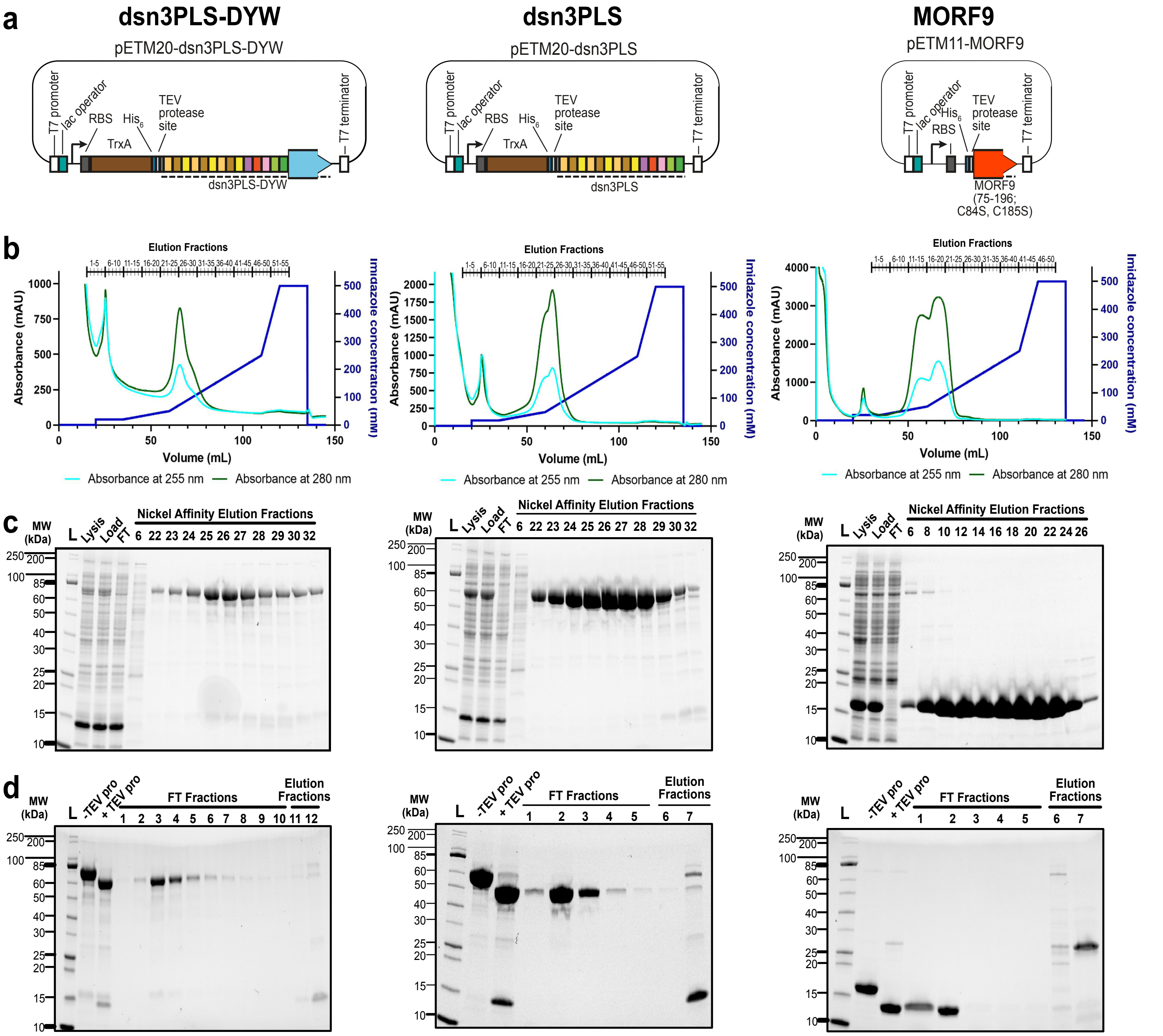

Supplementary Figure S2. Purification of dsn3PLS-DYW, dsn3PLS and MORF9.
a: Vector map of pETM20-dsn3PLS-DYW (left), pETM20-dsn3PLS (middle), and pETM11-MORF9 (right). **b**: Elution profiles from nickel affinity chromatography using imidazole (dark blue trace) as a competitor. Protein content was measured by absorbance at 255 nm (cyan trace) and 280 nm (dark green trace). **c**: Protein profiles of selected elution fractions. L, molecular weight ladder; Lysis, unpurified bacterial lysate; Load, fraction loaded on HisTrap column; FT, flow-through from column; 6-32, specific elution fractions numbered as in **b**. **d**: Reverse nickel-affinity purification of dsn3PLS-DYW, dsn3PLS and MORF9 following TEV cleavage of the N-terminal Ni-binding His-tag. L, molecular weight ladder; -TEV, control with no TEV added; +TEV, TEV cleavage products; FT fractions, flow-through fractions containing the desired unbound product; Elution fractions, fractions eluted from the column showing the bound, cleaved His-tag. Expected molecular weights for dsn3PLS-DYW, dsn3PLS, and MORF9 with their purification tags: 82.88 kDa, 68.39 kDa, and 17.22 kDa respectively. Expected molecular weights for dsn3PLS-DYW, dsn3PLS, and MORF9 without their purification tags: 68.64, 54.14, and 14.22 kDa respectively.

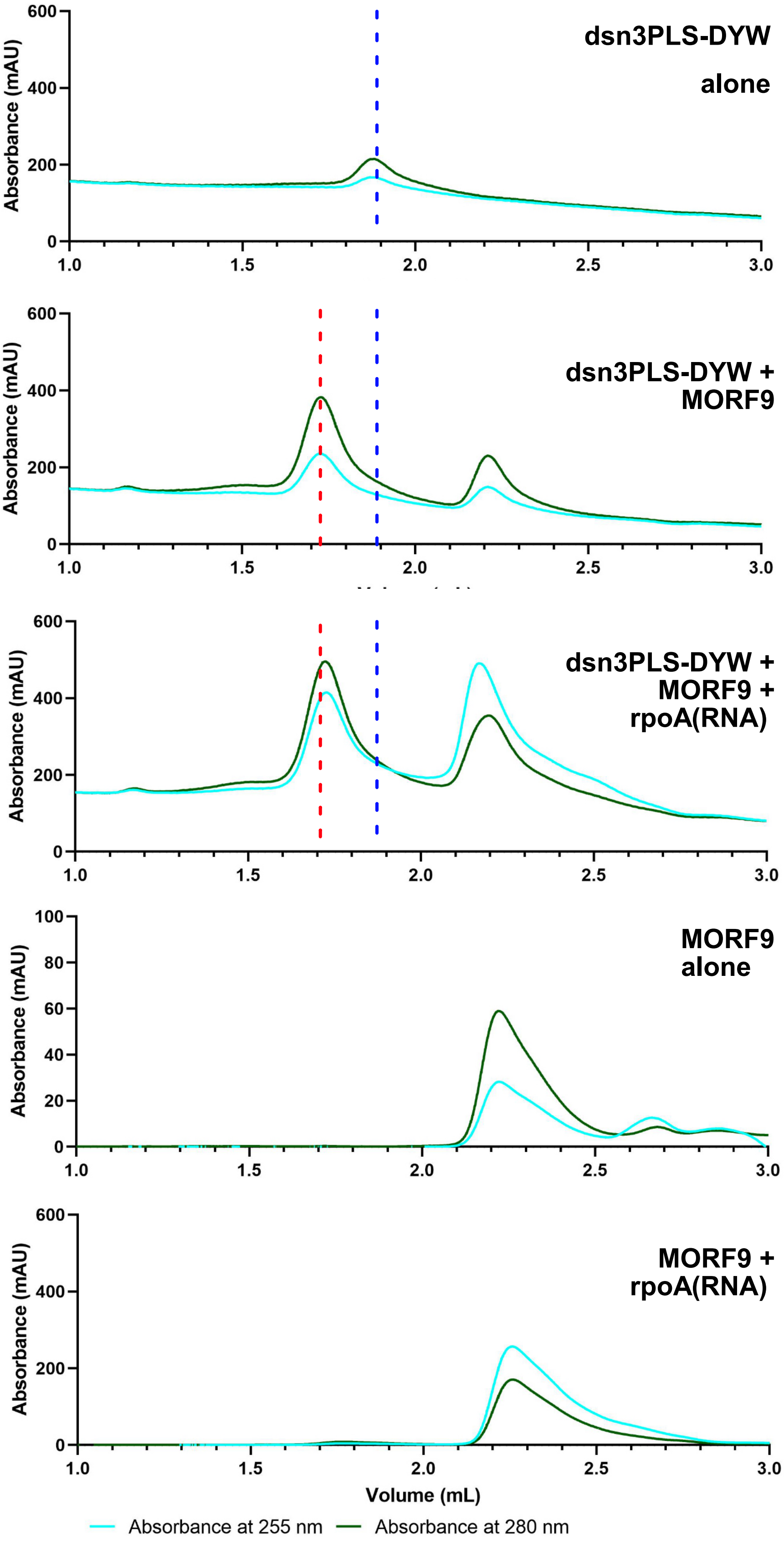

Supplementary Figure S3. Analytical size-exclusion chromatography of complexes between dsn3PLS-DYW, MORF9 and their RNA target.

Fractions leaving the column were analysed for protein and RNA content by absorbance at 280 nm and 255 nm respectively. The peak containing dsn3PLS-DYW (vertical blue dashed line) was shifted to a larger molecular weight complex (vertical red dashed line) by the addition of MORF9. The addition of the rpoA-78691 target oligonucleotide raised the absorbance at 255 nm of this peak, indicating the incorporation of the RNA into the high molecular weight complex. The samples shown contain 4.88 nmol of dsn3PLS-DYW, with 19.92 nmol of MORF9, and 10 nmol RNA oligonucleotide. This equates to a 4:1 ratio of MORF9 to dsn3PLS-DYW and a 2:1 ratio of RNA oligonucleotide to dsn3PLS-DYW.

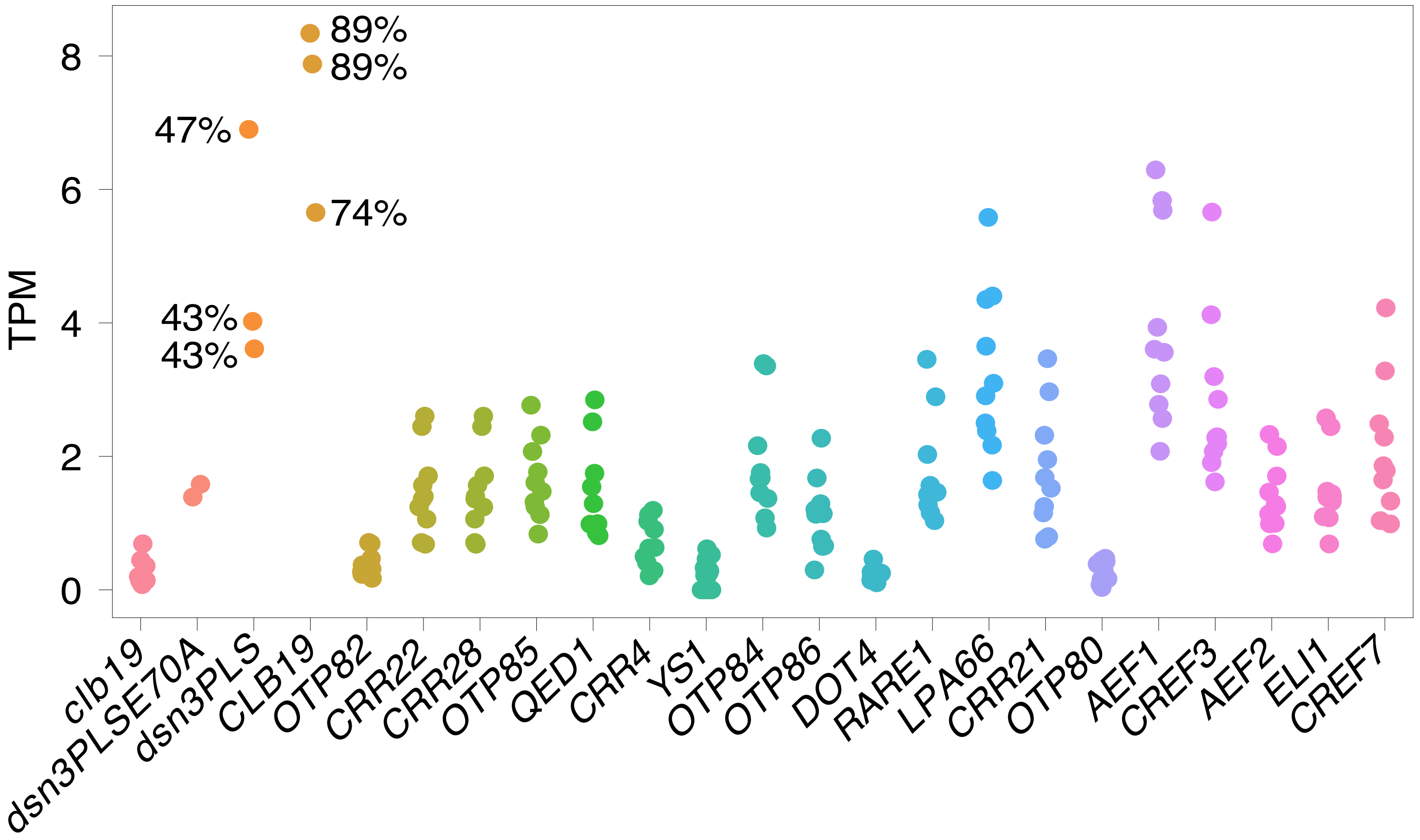

Supplementary Figure S4. Transcript abundance of editing factor transgenes and endogenous editing factor genes.

Transcript abundances were quantified as transcripts per million (TPM) by mapping RNA-seq reads to *Arabidopsis* transcripts supplemented with the sequences of the transgene constructs. For dsn3PLS-DYW and CLB19, the percentage editing at the *rpoA-78691* site is indicated for each of the transgenic lines to indicate how transcript abundance and editing are related.

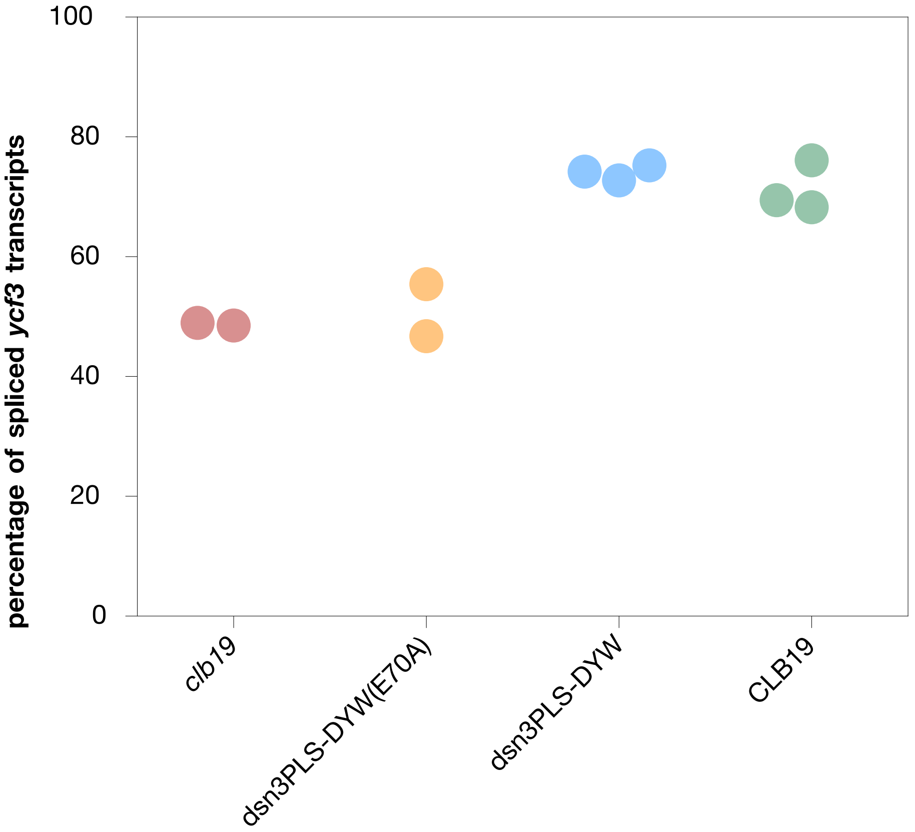

Supplementary Figure S5. Splicing of *ycf3* intron 2 is unaffected by editing of *ycf3-43350*. The extent of *ycf3* intron 2 splicing was estimated from RNA-seq data by counting reads crossing the donor (exon-intron) junction, the acceptor (intron-exon) junction and the spliced (exon-exon) junction. Percentage splicing was calculated as spliced/(spliced + (donor + acceptor)/2). The percentage splicing in plants expressing dsn3PLS-DYW (no editing of *ycf3-43350*) and CLB19 (~20% editing of *ycf3-43350*) is not statistically distinguishable (p-value 0.33, t-test, n=3 biological replicates).

dsn3PLS-DYW	CLB19	position	functional annotation	effect of editing
23/34518	**31384/36063**	**69942**	***clpP1* coding sequence**	**C**AU (His) -> **U**AU (Tyr)
**471/1071**	**1279/1575**	**78691**	***rpoA* coding sequence**	U**C**U (Ser) -> U**U**U (Phe)
2/3622	**829/4255**	43350	*ycf3* intron 2	
0/3810	**64/3940**	59002	intergenic (*accD-psaI*)	
**113/7898**	0/9704	114860	*ccsA* coding sequence	**C**UC (Leu) -> **U**UC (Phe)
**160/11446**	**99/12332**	118393	*ndhG* coding sequence	U**C**U (Ser) -> U**U**U (Phe)
**340/28513**	6/33229	116203	*ndhD* coding sequence	U**C**U (Ser) -> U**U**U (Phe)
1/1647	**19/1709**	9538	3' *atpA*	
0/2842	**37/3501**	111658	*ndhF* coding sequence	CU**C** (Leu) -> CU**U** (Leu)
5/19019	**139/22668**	14647	*atpI* coding sequence	**C**UU (Leu) -> **U**UU (Phe)
**76/13655**	1/16473	49646	*ndhK* coding sequence	**C**CU (Pro) -> **U**CU (Ser)
**149/26854**	1/30538	97693	*rps7* coding sequence	**C**AU (His) -> **U**AU (Tyr)
4/24888	**136/29717**	13561	5' *atpH*	
**49/9909**	1/11722	119998	*ndhA* coding sequence	**C**UU (Leu) -> **U**UU (Phe)
1/10196	**45/11786**	97277	3' *rps7*	
6/63955	**345/92791**	38871	*psaB* coding sequence	**C**CC (Pro) -> **U**CC (Ser)
0/36770	**122/39249**	66041	*petG* coding sequence	C**C**U (Pro) -> C**U**U (Leu)
**150/45178**	7/46938	69992	*clpP1* coding sequence	U**C**C (Ser) -> U**U**C (Phe)
**105/58902**	11/79032	39020	*psaB* coding sequence	U**C**A (Ser) -> U**U**A (Leu)
20/105478	**233/107140**	13258	3' *atpH*	
**69/102074**	25/114170	73588	*psbB* coding sequence	CU**C** (Leu) -> CU**U** (Leu)

Supplementary Table S1. Editing events detected in this study and their predicted functional impact.

The primary targets of CLB19 are indicated first, in bold. The other sites are presumed 'off-target' events. The values in the first two columns are editing events/read counts. Ratios in bold were statistically significant in comparison to the equivalent values from the negative control, i.e. clb19 plants expressing dsn3PLS-DYW (E70A). Where editing leads to a predicted amino acid change in the encoded protein, this is indicated in the final column, with the altered nucleotide in bold.

Oligonucleotide name	Sequence (5’ - 3’)	Modification	Purpose
*rpoA*_L	UAUUACACGUGCAAAAUCUG	None	Analytical size-exclusion chromatography
*rpoA*_REMSA	/5Cy5/AUGUAUUACACGUGCAAAAUCUGAGA	5′-Cy5 labelled	REMSA experiments
*clpP1*_REMSA	/5Cy5/CAGCAACAGAAGCCCAAGCUCAUGGA	5′-Cy5 labelled	REMSA experiments

Supplementary Table S2. RNA oligonucleotides used in this study.

Primer name	Sequence (5' – 3')	Purpose
SynthPLS.FOR	GAGAATCTTTATTTTCAGGGCGCCATGGGTAAT TCTGTTTTCTC	Cloning designed PPR proteins from first PLS triplet
SynthDYW.REV	TCAGTGGTGGTGGTGGTGGTGCTCGAGTTACC AATAATCTCCACAAG	Cloning designed DYW-type PPR proteins
DYW_E70A.FOR	GTTGTCTTACCATTCTGCGAAACTTGCTATCGC TTTC	Mutagenesis of catalytic E70 in the designer DYW domain
DYW_E70A.REV	GAAAGCGATAGCAAGTTTCGCAGAATGGTAAGA CAAC	Mutagenesis of catalytic E70 in the designer DYW domain
SynthSeq.FOR	ACTGTTGTTTCTTGGACAG	Sequencing designed PPR proteins
SynthSEQ2.FOR	GGTAGACTTGATGAGGCTTATGAG	Sequencing designed PPR protein cDNA from the S2 motif
SynthSEQ3.FOR	CTGGATATGTTCCTGATACTTC	Sequencing editing site in dsn3PLS-DYW
SynthSeq.REV	TCCGTAATGCTCAACTCTTG	Sequencing designed PPR proteins
T7short	TATGCTAGTTATTGCTCAG	Sequencing cDNA from the T7 terminator
*rpoA*_GA.FOR	AGGAACATGTATTACACGTGCAAAATCTGAGAG CTGAGCAATAACTAGCATAACCCCTTG	Insertion of *rpoA* editing site cassette
*rpoA*_GA.REV	GATTTTGCACGTGTAATACATGTTCCTTCTATTT CTCGAGTTACCAATAATCTCCACAAG	Insertion of *rpoA* editing site cassette
*clpP1*_GA.FOR	TTATGTCAGCAACAGAAGCCCAAGCTCATGGAG CTGAGCAATAACTAGCATAACCCCTTG	Insertion of *clpP1* editing site cassette
*clpP1*_GA.REV	GAGCTTGGGCTTCTGTTGCTGACATAAAAACAT CCTCGAGTTACCAATAATCTCCACAAG	Insertion of *clpP1* editing site cassette
clb19-3_RP	TTGATTTCTTGGACGGCTATG	Genotyping clb19-3 mutants
clb19-3_LP	GAGGAAGCGACAATGGTTATG	Genotyping clb19-3 mutants
LBb1.3	ATTTTGCCGATTTCGGAAC	Genotyping SALK T-DNA lines
MORF9_75.FOR	GGTGGTCCATGGAGCAGAGAGAGACGATTATG CTTCCTGGTTCCGATTAC	Cloning MORF9 75-196, introducing C85S mutation
MORF9_196.REV	GGTGGTCTCGAGTCACTGCTTTGGTTGGTATGT TGGGTATGTGGACGGGATAATCTC	Cloning MORF9 75-196, introducing C187S mutation
*rpoA*_ES.FOR	ATGGTTCGAGAGAAAGTC	Amplifying *A. thaliana rpoA* cDNA
*rpoA*_ES.REV	GATAAGGTCTTCTTGACTG	Amplifying *A. thaliana rpoA* cDNA
*rpoA*_PS.FOR	CAGTGGAAGTGTGTTGAATC	Sequencing *A. thaliana rpoA* editing site
*clpP1*_ES.FOR	TGCCTATTGGCGTTCCAAAAG	Amplifying *A. thaliana clpP1* cDNA

*clpP1*_ES.REV	TGAACCGCTACAAGATCAAC	Amplifying *A. thaliana clpP1* cDNA
*clpP1*_PS.FOR	GTATAGCATTCCCTCACGCTAG	Sequencing *A. thaliana clpP1* editing site
MORF2_73.FOR	GGTGGTCCATGGCGACGGAGATGGCTCCTTTG TTTCCGGGATCCGATTATG	Cloning MORF2 73-193, introducing C82S mutation
MORF2_193.REV	GGTGGTCTCGAGTCACTCAACCCGCCTCTGCC TCTCTGGTGATCGTTGGACTATC	Cloning MORF2 73-193

Supplementary Table S3. DNA primers used in this study.

Supplementary References

1. Yan, J. *et al.* MORF9 increases the RNA-binding activity of PLS-type pentatricopeptide repeat protein in plastid RNA editing. *Nat Plants* **3**, 17037 (2017).
